# Impact of Acute and Chronic Stress on Thrombosis in Healthy Individuals and Cardiovascular Disease Patients

**DOI:** 10.3390/ijms21217818

**Published:** 2020-10-22

**Authors:** Leonardo Sandrini, Alessandro Ieraci, Patrizia Amadio, Marta Zarà, Silvia Stella Barbieri

**Affiliations:** 1 Unit of Brain-Heart Axis: Cellular and Molecular Mechanisms, Centro Cardiologico Monzino IRCCS, 20138 Milan, Italy; patrizia.amadio@ccfm.it (P.A.); marta.zara@ccfm.it (M.Z.); 2 Laboratory of Neuropsychopharmacology and Functional Neurogenomics, Dipartimento di Scienze Farmaceutiche, Sezione di Fisiologia e Farmacologia, University of Milan, 20133 Milan, Italy; alessandro.ieraci@unimi.it

**Keywords:** psychological stress, acute stress, chronic stress, thrombosis, platelets, coagulation, endothelial dysfunction, inflammation

## Abstract

Psychological stress induces different alterations in the organism in order to maintain homeostasis, including changes in hematopoiesis and hemostasis. In particular, stress-induced hyper activation of the autonomic nervous system and hypothalamic–pituitary–adrenal axis can trigger cellular and molecular alterations in platelets, coagulation factors, endothelial function, redox balance, and sterile inflammatory response. For this reason, mental stress is reported to enhance the risk of cardiovascular disease (CVD). However, contrasting results are often found in the literature considering differences in the response to acute or chronic stress and the health condition of the population analyzed. Since thrombosis is the most common underlying pathology of CVDs, the comprehension of the mechanisms at the basis of the association between stress and this pathology is highly valuable. The aim of this work is to give a comprehensive review of the studies focused on the role of acute and chronic stress in both healthy individuals and CVD patients, focusing on the cellular and molecular mechanisms underlying the relationship between stress and thrombosis.

## 1. Introduction

Despite the thorough studies on the impact of classical risk factors on cardiovascular diseases (e.g., smoking, raised blood pressure levels, and high serum cholesterol levels) and the progress in diagnosis and treatment, these pathologies still remain the primary cause of morbidity and mortality, underlying the importance to find and characterize new risk factors.

Several studies linked chronic stress resulted from environmental noise, job strain, dementia caregiving, posttraumatic stress disorder, psychological distress from depressive and anxiety symptoms, and acute stress consequent to a psychological response to a terrifying, traumatic, or surprising experience to cardiovascular diseases (CVDs) [1,2,3,4,5].

This association drew the attention of the international societies of cardiology, leading the European Guidelines for Cardiovascular Disease Prevention to state that, in individuals with a recognized high risk or with an already established CVD, stress should be considered (suggested wording to use under the class of recommendation IIa) as a risk factor [6]. Interestingly, although the American Guideline on the Primary Prevention of CVD does not include stress among the risk factors, it is recommended for adults to be routinely assessed for psychosocial stressors and to be eventually provided with appropriate counseling [7].

Emerging data support the hypothesis that stress has only a modest effect in promoting the development of CVD in healthy individuals [1,2,8,9], whereas it might play a critical role contributing to a more severe progression and a worse clinical outcome in patients with a previous cardiovascular pathology [10,11,12,13,14].

In the MILIS study, emotional upset has been identified as potential trigger of myocardial infarction (MI) in most of 18% of patients enrolled [15], and a meta-analysis underlines that intense emotions increase the risk of MI by about 4.7 times [16]. In addition, both acute and chronic stress has been independently related to venous thromboembolism (VTE) [17,18,19].

Since thrombus formation plays a critical role in the pathophysiology of both MI and VTE, acute and chronic stress may promote these CVD complications predisposing to arterial thrombosis and venous thrombosis (VT), respectively.

Arterial thrombi have been historically characterized by the abundant presence of platelets and fibrin, and they occur in the setting of high shear flow and nearness of damaged atherosclerotic plaques. Venous thrombi have been considered rich in fibrin and red blood cells, occurring in an intact endothelial wall at places of slow shear flow. However, the strict structural difference between arterial and venous thrombi has been recently reconsidered. Both thrombi share a complex fibrin network containing platelets, leukocytes, and red blood cells, and it is only the different relative presence of these components that distinguish arterial and venous thrombi [20].

The underling mechanisms of thrombus formation may be summarized by the interaction among imbalance in the hemostatic system leading to hypercoagulability state, hemodynamic changes, endothelial dysfunction associated to inflammation, and oxidative stress. All these processes are observed in CVD patients and also in subjects under acute and chronic stress conditions. Then, the comprehension of the mechanisms by which stress can influence the players involved in the thrombotic process is highly valuable.

The aim of this work is to give a comprehensive review of the studies focused on the role of acute and chronic stress in both healthy individuals and CVD patients, focusing on the cellular and molecular mechanisms underlying the relationship between stress and thrombosis.

## 2. The Stress Responses

Stress is a natural and physiological reaction to challenging or threatening circumstances that promotes body adaptation and increases the chances to survive. The major pathways activated by stress are the autonomic nervous system (ANS) and the hypothalamic-pituitary-adrenal (HPA) axis. The ANS responds rapidly, within few seconds, following a stressful situation, whereas the HPA is involved in prolonged response.

During acute stressful experiences (short-term stress), all vertebrates put in place the so-called “fight or flight response” characterized by the release of chemical effectors, such as hormones and catecholamines, that promote changes in behavior, cardiovascular functions, endocrine and metabolic signals, and in immune response in order to maintain its homeostasis, a process named allostasis (literally meaning “achieving stability through change”) [21]. On the other hand, when the repetition of a build-up of stressors and stress-responses occur, the physiological changes described above lead to a maladaptive stress response that is known as “allostatic load” [22].

The capability to cope with stressful experiences differs among individuals, with the majority of people being able to successful adapt to challenging or threatening experiences, i.e., “resilients,” while others, i.e., “susceptibles,” have problem to properly adapt to stressful situations. Although the mechanisms underlying these different responses are not completely understood, it is assumed that the maladaptive response depends on the particular individual genetic background and/or previous life experiences [23,24,25].

### 2.1. Autonomic Nervous System

The ANS is divided in the sympathetic nervous system (SNS) and the parasympathetic nervous system (PNS), which promote opposite effects: the SNS stimulates the body’s “fight-or-flight” response, while the PNS activates the body to “rest and digest.” Under normal condition, a stressful experience triggers the activation of SNS that in turn promotes the production and release of catecholamines from the adrenal medulla. On the other hand, the PNS is activated when the stressful situation is diminished and, by releasing mainly acetylcholine, it plays a key role alleviating the stress response by inhibiting the SNS and HPA axis. However, during chronic stress, the sustained sympathetic activity and reactivity, which is not counteract by the PNS, results in enhanced cardiac tone, platelet activation and aggregation, coagulation, endothelial dysfunction, and inflammation [26]. The neuro-cardiovascular axis is formed by a series of interdependent afferent, efferent, and local neuronal circuits regulating the autonomic response to stress. The crossroad of the axis is the nucleus tractus solitarius (NTS) of the medulla, which receives afferences from different cortical regions, hypothalamus, peripheral chemoreceptors, and baroreceptors. The stimuli received in this region then modulate sympathetic and parasympathetic nuclei regulating autonomic nerve efferences and the activity of vagal innervation.

### 2.2. Hypothalamic–Pituitary–Adrenal (HPA) Axis

The HPA axis plays an important role in controlling and regulating the system that connects the central nervous system with the hormonal system. This stress-responsive neuroendocrine system helps the organism to adapt and maintain the homeostasis after challenges but is also vital in the normal physiological functions. In response to a stressful event, cortical areas of the brain are activated, and, through the limbic system, the signals arrive at the hypothalamus. Under neurotransmitter stimulation, cells located in the paraventricular nucleus (PVN) are activated to synthesize and secrete corticotropin-releasing factor (CRF) into the portal venous system connecting the hypothalamus and the pituitary gland [27,28]. In turn, the CRF stimulates the anterior pituitary gland to produce and secrete adrenocorticotropic hormone (ACTH) into the general circulation. Then, ACTH induces the production and release of glucocorticoids (GCs—cortisol and corticosterone in humans and rodents, respectively) from the adrenal cortex [29]. To protect against the deleterious effect of sustained activity, the HPA system is tightly modulated throughout negative-feedback loops designed to maintain physiological hormone levels and homeostasis. In particular, GCs inhibit activity of HPA by binding the glucocorticoid receptors (GRs) in the hypothalamus, hippocampus, pituitary, and medial prefrontal cortex (mPFC), resulting in a decrease in CRF secretion and subsequent reduced release of ACTH from the pituitary [27,28].

During the chronic stress response, cortisol is secreted continuously thus determining cortisol-resistance and a reduction in the negative feedback loop of the HPA. Cortisol and CRF were found to be able to induce endothelial dysfunction, thus participating in the onset and progression of plaque formation/rupture and coronary artery thrombosis [30].

## 3. Biological Processes and Molecular Mechanisms in Stress-Related Thrombosis

Although population-based observational surveys and clinical and experimental studies have established the association between stress and thrombosis, the underlying mechanisms of this relationship is not completely understood. Under stressful conditions, the organism implements an adaptive response that, through the activation of the HPA axis and the ANS, leads to the secretion of different glucocorticoid hormones and catecholamines (noradrenaline and adrenaline) [31] that directly or indirectly promote changes in the hemostasis. The enhanced platelet activation, endothelial dysfunction and coagulation, up-regulated inflammatory response, and altered fibrinolysis observed under stressful conditions play a pivotal role in the thrombotic processes associated to cardiovascular events.

### 3.1. Effect of Stress on Platelets

A large body of literature showed that stressful conditions stimulate thrombopoiesis and platelet activation [32,33,34,35,36,37]. The enhanced expression of glycoproteins, fibrinogen receptors, and P-selectin on the platelets surface promotes their aggregation and their interaction with leukocytes (platelet–leukocyte aggregate, PLA) in order to protect the organism from excessive bleeding in the fight-or-flight response during acute stress. In line with this information, it was observed that in healthy subjects the percentage of PLA returns to basal level between 20 and 45 min after an acute stress session, whereas it gradually increases until 75 min post-stress in CVD patients [38], indicating that, under stressful conditions, CVD patients are more prone to prolonged platelet activation compared to healthy subjects.

However, the impact of acute stress on platelet reactivity in healthy subjects produced contrasting results. Some studies showed an enhanced platelet activation in response to acute stress [35,36,39,40,41], whereas no significant changes in GPIb and GPIIb/IIIa expression on the platelet surface as well as in their fibrinogen binding ability in healthy subjects [42,43] or in platelet aggregation in animal models [44] were observed by others. It is reasonable to hypothesize that the variation in the methods used for the measurements and the different timing employed for platelet function assessment after stress may partially explain these discordant results.

More solid results have been found investigating the influence of acute stress on platelet function in CAD patients, where an adverse stressor may contribute to the predisposition of platelet hyper-reactivity [43,45]. Several studies showed that during acute mental stress, the platelet aggregation was enhanced in stable CVD and in post-myocardial infarct patients than in control subjects [43,45], and that the negative emotional state seems to be a crucial element predisposing to platelet activation in CVD. Indeed, a greater percentage of leukocyte–platelet, monocyte–platelet, and neutrophil–platelet aggregates have been measured in response to psychophysiological stress in coronary syndrome (ACS) patients undergoing an emotional trigger when compared to a non-triggered ACS [46]. In addition, a positive correlation between stress paradigms and platelet activation in terms of higher PLAs, greater platelet aggregation, enhanced expression of GPIIb/IIIa, P-selectin, and fibrinogen binding index was found in CVD patients [47,48].

Regarding the effect of chronic stress, it has been reported that low socioeconomic status (SES) and high work demand is accompanied by an enhanced platelet activation when compared to higher status colleagues or with samples taken during a more relaxing period, respectively [49].

Different animal models have definitely showed that continual and intensive stress increased platelet production and activation, enhancing the ability of thrombin and ADP to stimulate platelet aggregation and to promote platelet–leukocyte interaction [33,44]. The recognized mechanisms by which stressful conditions can affect platelet activation have been related to both HPA axis and ANS activation.

It is well established that platelet count, activation, and aggregation increased after exogenous administration of glucocorticoids to healthy volunteers [50,51] and that the same activation pattern is observed in patients with Cushing’s syndrome, a condition characterized by chronic hypercortisolism [52,53,54]. In line with these observations, the high cortisol levels measured during a period of high work demand have been associated with elevated thrombin-induced platelet aggregation [49], confirming a strong interplay among chronic stress, cortisol, and platelet function. According to the already known serotoninergic regulation of the HPA axis activity [55], a positive correlation between the concentration of platelets serotonin and cortisol levels was found in healthy subjects under stressful conditions. For this reason, the alterations of serotonin levels induced by stress could be crucial for their influence on platelet activation thus increasing the risk of coronary thrombosis [56].

Finally, both stressful conditions and catecholamines administration stimulate thrombopoiesis and increase the expression of GPIb and GPIIb-IIIa complex [35] and P-selectin [35,57], through the activation of α2-adrenergic receptors (α2-ADRs) widely expressed on platelets and megakaryocytes [35]. However, the higher levels of circulating catecholamine released under acute stress may induce the desensitization of α2-ADRs, as shown by the reduced receptor binding affinity in both human and animal platelets exposed to different stressors [58]. This condition can lead over time to an increased density of α2-ADRs on platelets [59] as a compensatory effect. Table 1 shows a summary of the effect of stress on platelets.

### 3.2. Effect of Stress on Coagulation and Fibrinolytic Cascade

Hemostasis includes a set of tightly regulated processes in which circulating coagulation proteins, platelets, and endothelium cooperate to maintain the delicate balance between prothrombotic and antithrombotic factors.

Traditionally, hemostasis is described as occurring in three phases: primary hemostasis, secondary hemostasis, and fibrinolysis. The interaction of platelets with activated endothelium leads to an unstable platelet plug (primary hemostasis), followed by activation of the coagulation system to form the fibrin clot (secondary hemostasis). The release of tissue factor (TF) by activated endothelial and smooth muscle cells, as well as by neutrophils and monocytes early recruited along with circulating microparticles, is the trigger of coagulation cascade [62].

TF, acting as a cofactor, promotes the proteolysis and activation of FVII (a) and formation of the extrinsic tenase complex (TF/FVIIa complex), which induces the activation of FIX and FX (FIXa and FXa). Then, FXa associated with cofactor FVa (prothrombinase complex) converts prothrombin (II) to thrombin (IIa) [63]. The slowly accumulating amounts of thrombin activates platelets adhesion to the site of injury, initiating the amplification phase, with further activation of FV (FVa) and conversion of FVIII into FVIIIa, which acts as a cofactor to FIXa on the surface of activated platelets. The tenase complex of FIXa/FVIIIa catalyzes the conversion of FX to FXa, which in turn forms the FXa/FVa complex producing sufficient amounts of thrombin to convert fibrinogen to monomer fibrin. As a final step, the thrombin-activated plasma transglutaminase FXIIIa catalyzes the formation of covalent crosslink between adjacent fibrin chains to yield an elastic, polymerized fibrin clot. This process is highly regulated to prevent uncontrolled widespread clot formation. Several anticoagulant factors (antithrombin, protein C and S system, thrombomodulin, tissue factor pathway inhibitor) inhibit or promote the degradation of activated coagulation factors [64,65,66].

Fibrinolysis is the last phase of hemostasis. The activation of plasminogen (PA) by serine proteases, including tissue type PA (tPA), urokinase type PA (uPA) and Kallikreinas as well as by FXIa and FXIIa, lead to plasmin formation, that accelerates the degradation of blood clots producing fibrin degradation products like D-dimer [67]. The over-regulation of plasmin or of plasminogen activator activity are prevented by plasminogen activator inhibitor 1 e 2 (PAI-1 and PAI-2), α2-antiplasmin (A2AP), and thrombin activable fibrinolysis inhibitor (TAFI) [68]. This fine balance may be overwhelmed by the overactivation of coagulation or impairment of fibrinolysis, leading to uncontrolled clot formation with consequent artery or vein occlusion.

An abnormal hemostatic response was found in healthy people and CVD patients under both acute and chronic stress conditions [35,69,70]. However, the mechanisms by which acute and chronic stressful conditions affect hemostasis are partially different. Under acute stress, both coagulation and fibrinolysis are concomitantly enhanced [18], although procoagulant factors increased more than fibrinolytic factors, resulting in a net prothrombotic state. Under chronic stress, only the procoagulant pathway is upregulated, while the fibrinolytic system is impaired [71]. In healthy subjects, psychologically acute stress increases plasma filtration with consequent blood hyperviscosity, decrease of plasma volume, increase of hematocrit, and activation of clotting factors [72,73].

Specifically, an increase of FVIIa, FVIIIa, FXIIa, fibrinogen, and von Willebrand factor antigen (vWF:Ag); thrombin–antithrombin complex (TAT); and D-dimer largely overcome the increase of t-PA determining a net hypercoagulability [18,30,72,74,75,76,77,78,79]. In addition, activation of the extrinsic and intrinsic coagulation pathways was observed, as shown by the increase of the percent prothrombin time (PT) and decrease in activated partial thromboplastin time (aPTT) [73,80], even if two studies failed to demonstrate a change in aPTT levels [81,82].

Interestingly, age and sex also seem to play a role in the stress response in healthy subjects. A positive association between increased age and greater fibrinogen, D-dimer, or FVIIa levels was found [81]. Age and stress interact to modulate D-dimer levels, and this relationship subsists both immediately and 20 min after stress session [83]. Of note, FVIIa activity increased only in men exposed to acute stress, whereas in women a significant increase in activity of t-PA activity was found [79,84].

Similarly, CVD patients exposed to acute stress displayed an abnormal hyper-activation of coagulation cascade. Higher D-dimer [85], delayed recovery of consumed antithrombin III [86], and reduced activation of the fibrinolytic pathway [87] were found in these patients compared to healthy controls.

As already reported, under chronic stress conditions the increased procoagulant activity (e.g., high fibrinogen, D-dimer, FVIIa, FVIIIa, and vWF:Ag) is associated with the impairment in the fibrinolytic pathway, as showed by the increase of PAI-1 expression and activity and a decrease in t-PA activity [18]. However, the paradigm of chronic stress used could affect hemostasis differently. Both low socioeconomic status and job stress were associated with elevated levels of fibrinogen, FVIIa, FVIIIa, vWF, D-dimer, and PAI-1 [49,72,88,89,90,91]. In addition, work-related stress reduced t-PA activity without affecting PT and aPTT [72,88,89]. By contrast, caregiver stress was mainly associated with increased levels of vWF:Ag, PAI-1:Ag, TAT, D-dimer, and t-PA:Ag [92,93,94] and a massive increase of D-dimer level [92,93,95,96], which remain higher than in healthy subjects up to 30 months after the end of caregiving [60].

Chronic stress could participate to the alteration of the hemostatic system through different mechanisms. Focusing on the main stress pathway (HPA axis and SNS), chronic administration of corticosteroid or hypercortisolism (e.g., Cushing’s syndrome) enhanced plasma levels of vWF, PAI-1 and FVIII levels, and decreased markers of fibrinolytic activation [50,97,98,99,100,101,102]. Similarly, the administration of glucocorticoids in healthy subjects increased levels of fibrinogen and vWF [50,101,102,103], whereas during active inflammation, there were increased levels of PAI-1 but concomitantly decreased levels of VWF and fibrinogen [104], reducing inflammation and the pulmonary embolism in trauma patients [105].

It was observed that acute mental stress can trigger a hypercoagulable state, increasing circulating catecholamines level and β2-adrenergic receptor (β_2_-AR) sensitivity [74]. The sympathetic stimulation in arteries and arterioles induces adrenaline release, stimulating in a dose-dependent manner the vascular endothelial cells through the activation of β_2_-ARs, which promotes the release of FVIII, vWF, and t-PA from endothelial into the circulation [35,106]. Similarly, infusion of adrenaline or selective and nonselective β_2_-AR agonists (salbutamol and isoproterenol or propranolol, respectively) elicits procoagulant state, shortening aPTT, enhancing FV and FVIII and vWF levels, and increasing in D-dimer and prothrombin 1+2 fragments [107,108,109]. Interestingly, these effects were not observed after administration of noradrenaline, or α-ADR and β1-ADR agonist [35,74,108,110]. These observations provide evidence of the key role of β_2_-ADR activation on coagulation. Accordingly, the nonselective β-ADR antagonist propranolol abolished the release of FVIII induced by adrenaline [108] and reduced FVIII levels in patients with DVT [109]. Table 2 and Table 3 show a summary of the effect of acute and chronic stress, respectively, on coagulation and fibrinolysis.

### 3.3. Effect of Stress on Endothelial Dysfunction

The endothelium can be considered a dynamic organ in which endothelial cells, responding to neurotransmitters and vasoactive factors, maintains control of the vascular function including vasomotion and endothelial barrier integrity [114].

The dynamics of vascular perfusion and shear stress are managed by endothelial-derived vasodilatative and vasoconstrictive factors resulting in a balance of vascular relaxation and contraction. In endothelial dysfunction, homeostasis of vessel resistance is impaired, leading constrictive factors to prevail over vasodilative factors. A central vasodilative role is played by NO and by prostacyclin, while endothelin-1 (ET-1) and prostanoids—including thromboxane A2 (TXA2), prostaglandins D2, E2, and/or F2α derived from arachidonic acid by the activation of cyclooxygenases (COX-1 and COX-2) enzymes—are the main vasoconstrictive molecules. Interestingly, the neurotransmitter acetylcholine (Ach) plays a dual role: in physiological conditions, it acts as a vasodilator, enhancing NO release from endothelial cells; whereas in pathological conditions, it acts as vasoconstrictor due to a direct effect on smooth muscle cells.

Indeed, when pathological conditions occur, mechanical stress, inflammation, and oxidative stress [115,116,117] perturb the physiological endothelial response resulting in endothelial dysfunction, a first step of vascular disease.

In the last few years, different techniques have been developed to assess endothelial function in vivo (reviewed in references [118,119]). Non or less invasive surrogate techniques compared to the invasive functional coronary angiogram are usually preferred to investigate endothelial function to avoid the considerable risk for the patient. Among these techniques, forearm flow-mediated vasodilatation (FMD) is easy to perform and well correlated with coronary endothelial function measurement [120,121,122]. For this reason, studies in the last 20 years have focused on FMD measurements, showing a relationship between stress and enhanced endothelial dysfunction.

The studies carried out to investigate the impact of acute stress on endothelium have produced conflicting results. In some studies, acute mental stress increased forearm blood flow and induced FMD in healthy subjects in a sex independent manner [123,124]. By contrast, other authors provided evidence that acute psychological stress was associated to a systemic vasoconstriction and that the FMD was still reduced within 30–90 min after stress session [125,126,127].

Despite few studies investigated the relationship between chronic stress and vascular function, the results produced support its role to promote endothelial dysfunction. Impaired vasodilation was observed in response to chronic stress and sleep deprivation [128], mood disturbance [129], and in subjects perceiving themselves to be of lower social status in their communities [130], whereas no association was found between other subjective or objective social socioeconomic status (e.g., education, job) and FMD alterations [130,131]. In caregivers, an association between stress and endothelial dysfunction was observed only when considering the time span of the stressor [132,133] but not when considering the self-reported stress level [132].

However, a large sample size may be necessary to reveal some differences as suggested by the recent multi-ethnic study of middle- and older-age adults performed in 2963 people. Interestingly, in this study, chronic stress was associated with reduced FMD independent of sociodemographic characteristics, and this relationship persisted after adjustment for blood pressure, waist circumference, and cholesterol [134].

To our knowledge, there are no studies investigating the effect of psychological stress on endothelial dysfunction in CVD patients. For this reason, we encourage research in these people in order to deepen the knowledge in high risk CVD patients. Dysfunctions in SNS, HPA axis, and nitric-oxide synthase (NOS) activity might underlay the relationship between stress and endothelial dysfunction. The role of cortisol in stress response has been extensively explored to understand not only its importance in stress-mediated endothelial dysfunction but also the potential mediators of the observed effects. Elevated levels of cortisol are associated with reduced FMD induced by Ach, as observed in Cushing’s syndrome patients and in healthy subjects after oral administration of cortisol when compared to control [135,136]. Interestingly, in the general population, when the cortisol production, induced by a stress task, was blocked by administration of metyrapone, FMD was impaired [137].

In vitro [138] and in vivo [139] studies showed that cortisol could contribute to stress-mediated endothelial dysfunction inhibiting the expression of eNOS interacting with the suppressive glucocorticoid response element present in the eNOS promoter region [140]. It was also suggested that cortisol may modulate NO availability, increasing production of reactive oxidative species (ROS) [141], as provided by the administration of vitamin C, an anti-oxidant drug, that restored the abnormal blood flow to glucocorticoid induced pre-stress level [142]. Finally, cortisol counteracts the NO-mediated vascular function promoting the direct release of ET1, a potent vasoconstrictor, from SMC [143] and endothelium [144]. The critical role of ET1 in stress-related endothelial dysfunction was demonstrated in healthy people. Acute stress increased the circulating levels of ET1 [145,146], and the administration of ETA receptor antagonist abrogated the reduction of FMD metal stress-induced [147].

No robust evidence of the role of SNA in the modulation of endothelial function has been produced. Intra-arterial infusion of noradrenaline did not affect FMD, suggesting that catecholamine release did not interfere with endothelial NO function [147]. However, Hijmering et al. [148] showed that the reduction in FMD induced by the direct activation of SNA was restored blocking the α-ADR with phentolamine. Interestingly, Eriksson et al. provided evidence that, after an acute mental stress, challenge impairment in resistance vessel endothelial function is sustained by β-ADR receptor (and not α-ADR receptor) [149]. In line with this evidence, a recent meta-analysis demonstrated that in patients taking β-blockers, the sympathetic effect on the vascular system and the improvement of endothelial dysfunction is reduced compared to placebo [150].

The different approaches used to induce SNA can lead to different activation of the muscle sympathetic nerve activity [151] or long-lasting effects mediated by sympathetic activation on heart rate and blood pressure [127,137], which might explain the contrasting results found in the literature.

### 3.4. Effect of Stress on the Sterile Inflammation Response

Besides the canonical inflammatory response associated to infection, the occurrence of mechanical trauma and ischemia, leading to cellular necrosis and damage associated molecular patterns (DAMPs) response, or the presence of toxins, minerals, crystals, chemicals, and antigens trigger the so-called sterile inflammatory response. During sterile inflammation, the dominant effect is the collateral damage to the healthy cells of a specific tissue (e.g., gout) or to several body districts, which trigger acute disease and/or exacerbate the damage already present for previous pathological conditions [152].

During sterile inflammation, as occurring during a canonical inflammatory response, the body responses by promoting vasodilation associated with fluid leakage in order to deliver antibodies, cytokines, chemokines, and leukocytes to the site of injury. Once the inflammatory stimulus is neutralized, the site is cleared, and tissue repair can begin.

The sterile inflammation response has been recently proposed to explain the association between inflammation and psychological stress. Of note, the concept of sterile inflammation applied to stress-associated inflammation has a double and opposite effect. On one hand, stress-associated inflammation is useful to prime immunity in response to injury [153]; on the other, it can increase the inflammation, in particular enhancing the blood levels of DAMPs and cell death, in already existing disease conditions, thereby worsening the prognosis [154]. Stressful events can trigger immune response leading to inflammation both in the CNS and in other organ systems [155]. In the last 20 years, several studies established that the activation of the immune response, with enhanced circulating levels of CRP, IL-6, IL-1β, TNF-α, etc., can contribute to the development and progression of CVD [156].

Several studies have found that acute stress exposures are followed by increase in plasma levels of inflammatory markers. A recent meta-analysis by Marsland et al. [157], which updated the previous analysis performed by Steptoe et al. [158], found a significant stress-related increase in IL-6 and IL-1β, TNF-α, IL-2, and IL-10 but did not confirm the significant increase of C-reactive protein (CRP) previously reported. No differences in IL-1ra, IFN-γ, IL-4, IL-8, and IL-12 levels were found. These results could be related to the low number of studies measuring these cytokines, as well as to the post-stress time of sample collection, and to the association of psychosocial background factors. Circulating levels of these cytokines are enhanced within 10–40 min post stress (IL-6, TNF-α, IL-1β, IL-2, or IL-10) and progressively increase until 90 or 120 min post-stress. Further experiments, aimed at the study of cytokines dynamics after stress might follow the subject for more than two hours, are needed to define the peak-time and the recovery of cytokines. In addition, the levels of cytokines measured following an induced laboratory acute stress are highly influenced by anger or anxiety during the stress test [159] or by pre-existing depressive status [160,161], self-esteem [162], loneliness [163], socioeconomic status [164,165], or stress work [166].

Even if sterile inflammation is recognized to contribute in the development and progression of CVD [133], to our knowledge, there are no studies regarding the impact of psychological stress on the inflammatory profile of CVD patients. For this reason, we encourage research on this field to better understand this relationship in people with an already existing CVD history.

Activation of the HPA axis and/or the ANS have been proposed as mechanisms to explain the impact of stress on peripheral inflammatory markers.

A positive correlation between the activation of ANS and the pro-inflammatory markers was found [153,167]. In particular, short-term sympathetic response might increase circulating noradrenaline levels and enhance macrophage recruitment by the activation of β-ADR [151,167,168]. On the contrary, an inverse correlation between circulating inflammatory molecules and the release of cortisol induced by the activation of HPA axis, in line with the well-known anti-inflammatory properties of this molecule, was found [167,169,170,171].

Under intensive chronic stressful conditions, IL-6, CRP, and TNF-α are altered in every enduring adverse psychosocial condition considered. In particular, IL-6 plasma levels are higher in caregivers compared to the general population [95,172,173]. In addition, a positive correlation between the time spent to caregiving and IL-6 level was found in independent follow-up studies at 1–6 years, suggesting an acceleration in the accumulation of plasmatic inflammatory markers directly related to age [174,175]. Similarly, low SES and being part of a visible minority ethnic group correlated with higher levels of IL-6 and CRP in different pooled and single cross-sectional analyses [176,177,178]. Higher CRP is also found in many but not all the studies [95,179,180], and this increase is long-lasting; indeed, a significant reduction of CRP level was found three months after the death of the spouse [181]. Finally, work-related stress inflammation was mainly studied in burnout syndrome, a frequent consequence of work overload during the time. In this condition, high levels of TNF-α [182,183], IL-6 [183], and CRP were found [184,185], with the exception of one study [186].

Long-term activation of HPA axis has been proposed as the leading cause of inflammatory response during chronic stress conditions. In particular, the prolonged release of cortisol is related to cortisol dysfunction [187], determining cortisol depletion, impaired secretion, insufficient CRF function, GR resistance or down-regulation, and failure of negative feedback system [188].

Interestingly, in the last few years, stimulation of stress-induced sterile inflammation generated by damage-associated molecular patterns (DAMPs), and its regulation by miRNA and exosomes have emerged (reviewed in [189]). The core concept is that stressful conditions are able to induce DAMPs concentration in blood (e.g., Hsp72), increase pro-inflammatory proteins associated with blood-borne exosomes (e.g., Hsp72), and reduce anti-inflammatory intra-exosomal miRNA (e.g., let-7 miRNA family) [190]. The use of an α-ADR antagonist [189] reduced all the stress-associated alterations described above and let us hypothesize that stress-induced SNA could mediate inflammatory response also by DAMPs, miRNA, and exosomes alterations.

All these findings must be taken into account in order to design future studies aimed at the comprehension of the real driving force, stress response per se, or psychosocial background factors involved in the acute stress-induced inflammatory response. Table 4 shows a summary of the effect of acute and chronic stress on inflammatory markers.

### 3.5. Effect of Stress on Oxidative Imbalance

Oxidative stress may by narrowly summarized as the imbalance between production and clearance in the levels of reactive oxygen species (ROS) and nitrogen species. Different studies clearly demonstrated that different pathologies, including CVD, are associated or even triggered by them [191]. Evidence suggests that the association among oxidative stress, inflammation, and endothelial dysfunction underlie the pathophysiology of CVD morbidity and mortality [192,193].

In particular, the imbalance between oxidant and anti-oxidant enzymes plays a critical role in the development of thrombotic process [194,195,196]. In particular, it was demonstrated that, among the factors involved in thrombus formation, ROS enhanced the expression of TF associated to platelets and mononuclear cells [197,198,199], and that oxygen radicals upregulated the expression of PAI-1 in endothelial cells [200]. In order to maintain hemostasis, the pro-thrombotic effect mediated by ROS can be buffered by NO released from both platelets and endothelium [201,202]. Interestingly, it was found that platelets from ACS patients displayed enhanced ROS production and an impaired release of NO [203].

Disturbed redox homeostasis has been proposed as one of the molecular link between stress and CVD [204], and evidence suggested that brain O_2_^−^ is critical for stress-mediated cardiovascular response [205]. However, whereas the effect of acute stress in the brain is well established [206], its impact on direct and indirect biomarkers of oxidative stress in circulating cells and biological fluids produced controversial results. Acute stress reduced the generation of ROS and of superoxide anion in healthy subjects [207,208], whereas increased ROS generation affected the enzymatic antioxidant system in an animal model [209,210,211,212]. Although few studies clearly addressed the relationship between chronic stress, circulating oxidative markers, and CVD, chronic stress is accepted as a mechanism able to worsen oxidative stress. In animal models of chronic stress, increased lipid peroxidation, ROS production, malondialdehyde levels, and reduced activity of antioxidant system enzymes were found [213], thus predisposing to endothelial dysfunction [214] and thrombosis [33]. In humans, job stress was associated to an increase in urinary hydrogen peroxide and 8-hydroxy-2′-deoxyguanosine [215]. In chronically stressed woman undergoing an acute stress test, anticipatory cortisol reactivity was associated with increased levels of 8-iso-prostaglandin F2α and 8-hydroxyguanosine [216]. While the role of oxidative stress in CVD is well established [217], controlled studies are needed to clearly understand the possible role of acute and chronic stress in the development of CVD through oxidative stress pathways [204].

## 4. Conclusions

Studies and meta-analyses published in the last years clearly suggest that stress is an important risk factor for cardiovascular diseases. For this reason, cardiovascular societies recognize that more efforts are needed to clearly understand the cellular and molecular mechanisms by which psychological factors can influence cardiovascular disease in order to identify potential treatments. In addition, psychological prevention and management strategies should be introduced in clinical practice, with particular attention for patients with pre-existing CVD.

All the data from the literature did not provide unequivocal evidence that both acute and chronic stress alone suffice to provoke acute coronary thrombosis events, whereas they strongly suggest that in presence of other risk factors stress may confer a greater risk for CVD. Stress response determines a pro-thrombotic state characterized by autonomic and neuroendocrine dysfunction, platelet activation, dysregulation of coagulation, fibrinolysis, and endothelial dysfunction and inflammation. However, this physiological stress response can be considered a pathological trigger only in susceptible patients, worsening the prognosis and the outcome in the ones with pre-existing CVD. Starting from this evidence, Burg et al. [218] proposed the conceptual model of a “perfect storm” in which only the co-occurrence of different pathophysiologic and psychosocial factors, each acting in tandem with one another, can explain the increased risk induced by stress. The field of behavioral cardiology should take into account this concept in order to address this complex interplay in high risk patients. This could help to develop screening and intervention programs to put in place prevention strategies improving patient management. While some small studies demonstrated that behavioral interventions such as relaxation, emotion regulation, or medications can help to reduce pro-thrombotic responses to stress, more controlled studies are necessary to clearly investigate the real effectiveness of these strategies. These studies might help to provide clearer guidelines in order to improve the management of stress in CVD patients reducing the onset and progression of the pathology.

## Figures and Tables

**Table 1:** Alteration of platelets according to stress type and subjects’ status.

Parameter	Effect on Thrombosis	Stress Type	Subjects Status	Variation
GP-Ib	Increased levels lead to higher platelet adhesion	Acute	Healthy	↑ [35]
		Acute	Healthy	= [42,57]
		Acute	Stable angina	= [43]
Gp IIb-IIIa complex	Increased levels lead to higher platelet activation	Acute	Healthy	↑ [35]
		Acute	Healthy	= [42,43]
		Acute	Stable angina	= [43]
		Acute	CAD and CHD	↑ [46,47,48]
P-selectin	Increased levels lead to higher platelet adhesion to the surface of activated endothelial cells and higher activated platelets	Acute	Healthy	↑ [35,57]
		Acute	CAD and CHD	↑ [46,47,48]
		Chronic-Caregivers	Healthy	↑ [60,61]
Platelet-leukocytes aggregates	Increased levels are considered a marker of prothrombotic state	Acute	Healthy	↑ [35,40]
		Acute	CAD	↑ [45]
Platelets aggregates	Increased levels facilitate arterial thrombus formation	Acute	Healthy	↑ [38]
		Acute	CAD	↑ [44]
		Chronic- Low SES	Healthy	↑ [48]
Platelets count	Increased levels lead to higher thrombotic risk	Acute	Healthy	↑ [35]

GP, Glycoprotein; ↑, increased; =, unvaried.

**Table 2:** Alteration of coagulation and fibrinolysis factors caused by acute stress.

Acute Stress		
Parameter	Analyzed Parameter	Variation
Coagulation	FVIIa	↑ [72,76,79]
	FVII:Ag	= [111]
	FVIIIa	↑ [72,76]
	FVIII:Ag	= [111]
	FXIIa	↑ [72,76]
	FXII:Ag	= [111]
	Fibrinogen	↑/= [79]/[72]
	vWF	↑ [20,72,76,78]
	TAT	↑ [78]
	D-dimer	↑ [20,76,78]
	% PT	↑ [73,80]
	aPTT	↓/= [73,80]/[81,82]
Fibrinolysis	t-PAa	↑ [20,76,78,79,112]
	PAI-1 activity	= [79,112]
	PAI-1:Ag	↓ [113]

F, factor; a, clotting activity; ag, antigen; vWF, von Willebrand Factor; TAT, thrombin–antithrombin complex; PT, prothrombin time; aPTT, activated partial thromboplastin time; t-PA, tissue-plasminogen activator; PAI-1, plasminogen activator inhibitor-1. ↑, increased; ↓, decreased; =, unvaried.

**Table 3:** Alteration of coagulation and fibrinolysis factors caused by chronic stress.

Chronic Stress			
Parameter	Analyzed Parameter	Stress Domain	Variation
Coagulation	FVIIa	Low SES	↑ [88,89]
		Job stress	↑ [49,90,91]
	FVIIIa	Job stress	↑ [49,90,91]
	Fibrinogen	Low SES	↑ [88,89]
		Job stress	↑ [49,90,91]
	vWF	Caregivers	↑ [92,93,94]
	TAT	Caregivers	↑ [92,93,94]
	D-dimer	Low SES	↑ [88,89]
		Caregivers	↑ [92,93,94]
	% PT	Job stress	= [49,90,91]
	aPTT	Job stress	= [49,90,91]
Fibrinolysis	t-PAa t-PA:Ag PAI-1 activity	Job stress	↓ [49,90,91]
		Caregivers	↑ [9,92,93,94,112]
		Low SES	↑ [88,89]
		Job stress	↑ [49,90,91]
		Caregivers	↑ [92,93,94]

F, factor; a, clotting activity; ag, antigen; vWF, von Willebrand Factor; TAT, thrombin–antithrombin complex; PT, prothrombin time; aPTT, activated partial thromboplastin time; t-PA, tissue-plasminogen activator; PAI-1, plasminogen activator inhibitor-1. ↑, increased; ↓, decreased; =, unvaried.

**Table 4:** Alteration of inflammatory markers factors during acute and chronic stress.

Parameter	Acute	Chronic
IL-1β	↑ [159,160]	?
IL-2	↑ [159,160]	?
IL-6	↑ [159,160]	↑ [95,174,175,176,177,178,179,180,185]
IL-10	↑ [159,160]	?
TNF-α	↑ [159,160]	↑ [95,174,175,184,185]
CRP	↑ [160]	↑ [178,179,180,186,187]
	= [159]	= [95,181,182]
IL-8	= [159,160]	?
IL-12	= [159,160]	?
IL-4	= [159,160]	?
IL-1ra	= [159,160]	?
IFNγ	= [159,160]	?

IL, interleukin; TNF-α, tumor-necrosis factor-α; CRP, C-reactive protein; IFN-γ, interferon-γ. ↑, increased; =, unvaried; ?, unknown.

## Abbreviations

Ach	Acetylcholine
ACTH	Adrenocorticotropic Hormone
Ag	Antigen
ANS	Autonomic Nervous System
aPTT	Activated Partial Thromboplastin Time
a	Clotting activity
CRF	Corticotropin-Releasing Factor
CRP	C-Reactive Protein
CVDs	Cardiovascular Diseases
DAMPs	Damage Associated Molecular Patterns
ET1	Endothelin-1
ETA	Endothelin receptor A
F	Factor
FMD	Flow-Mediated Dilation
GCs	Glucocorticoids
GP	Glycoprotein
GRs	Glucocorticoid Receptors
HPA	Hypothalamic–Pituitary–Adrenal
Hsp	Heat-shock protein
IFN-γ	Interferon-γ
IL	Interleukin
MI	Myocardial infarction
miRNA	microRNA
mPFC	medial Prefrontal Cortex
NTS	Nucleus Tractus Solitarius
PAI-1	Plasminogen Activator Inhibitor-1
PLTs	Platelets-Leukocyte aggregates
PT	prothrombin time
PVN	Paraventricular Nucleus
ROS	Reactive Oxygen Species
SES	Socio-Economic Status
SMC	Smooth Muscle Cells
TAT	Thrombin–Antithrombin Complex
TNF-α	Tumor-Necrosis Factor-α
t-PA	tissue-Plasminogen Activator
t-PA:Ag	tissue-Plasminogen Activator Antigen
VT	Venous Thrombosis
VTE	Venous Thromboembolism
vWF	von Willebrand Factor
vWF:Ag	von Willebrand Factor Antigen
α2-ADRs	α2-Adrenergic Receptors
α-ADR	α-Adrenoreceptor
α-MSH	alpha-Melanocyte-Stimulating hormone
β2-ADR	β2-Adrenergic Receptor
β–ADR	β-Adrenoreceptor
